# The identification and semi-quantitative assessment of gastrointestinal nematodes in faecal samples using multiplex real-time PCR assays

**DOI:** 10.1186/s13071-021-04882-4

**Published:** 2021-08-09

**Authors:** Nikol Reslova, Lucie Skorpikova, Iveta Angela Kyrianova, Jaroslav Vadlejch, Johan Höglund, Philip Skuce, Martin Kasny

**Affiliations:** 1 Department of Botany and Zoology, Faculty of Science, Masaryk University, Brno, Czech Republic; 2 Department of Zoology and Fisheries, Faculty of Agrobiology, Food and Natural Resources, Czech University of Life Sciences Prague, Prague, Czech Republic; 3 Department of Biomedical Sciences and Veterinary Public Health, Section for Parasitology, Swedish University of Agricultural Sciences, Uppsala, Sweden; 4 Moredun Research Institute, Pentlands Science Park, Edinburgh, UK

**Keywords:** Gastrointestinal nematode, Sheep, Multiplex detection, Real-time PCR, Cell-free DNA

## Abstract

**Background:**

The diagnosis of gastrointestinal nematode (GIN) infections in ruminants is routinely based on morphological/morphometric analysis of parasite specimens recovered by coprological methods, followed by larval culture (LC) techniques. Such an approach is laborious, time-consuming, requires a skilled expert, and moreover suffers from certain limitations. Molecular tools are able to overcome the majority of these issues, providing accurate identification of nematode species and, therefore, may be valuable in sustainable parasite control strategies.

**Methods:**

Two multiplex real-time polymerase chain reaction (PCR) assays for specific detection of five main and one invasive GIN species, including an internal amplification control to avoid false-negative results, were designed targeting *SSU rRNA* and *COI* genetic markers, as well as established *ITS1/2* sequences. The assays were optimized for analysis of DNA extracted directly from sheep faeces and verified for *Haemonchus contortus*, *Teladorsagia circumcincta*, *Trichostrongylus colubriformis*, *Nematodirus battus*, *Chabertia ovina*, and *Ashworthius sidemi*. Semi-quantitative evaluation of infection intensity was enabled using a plasmid construct and a dilution series of sheep faeces with a known number of nematode eggs. Assays were tested on 44 individually collected faecal samples from three farms, and results were compared to those from faecal egg counts (FEC) using the concentration McMaster technique and LC.

**Results:**

Multiplex real-time PCR assays showed great specificity to target nematodes. During the analysis of faecal samples, the assays proved to have higher sensitivity in strongylid-type egg detection over FEC by revealing three false-negative samples, while showing moderate agreement in evaluation of infection intensity. The multiplex assays further clarified GIN species identification compared to LC, which had confused determination of *Teladorsagia* spp. for *Trichostrongylus* spp.

**Conclusions:**

Our multiplex assays proved to be a rapid and accurate approach enabling simultaneous and reliable GIN species identification from faeces and semi-quantitative estimation of the number of eggs present. This approach increases diagnostic value and may add a high degree of precision to evaluation of anthelmintic efficacy, where it is important to identify species surviving after treatment.

**Graphical Abstract:**

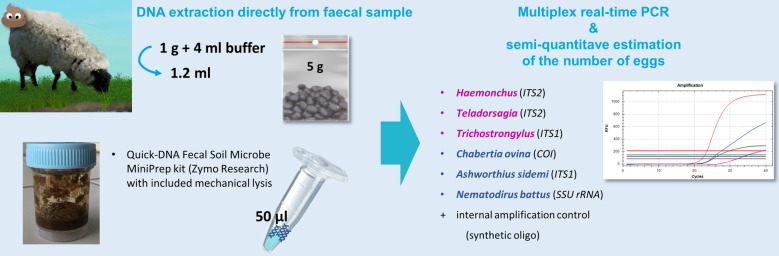

**Supplementary Information:**

The online version contains supplementary material available at 10.1186/s13071-021-04882-4.

## Background

All grazing livestock are exposed to gastrointestinal nematode (GIN) infections. The higher the worm load, the greater the impact on animal health, productivity, and welfare [1]. Typically, grazing ruminants are infected with several species of GINs whose unique mix contributes to the sum of the clinical effects and determines the severity of infections. In combination, these infections are responsible for parasitic gastroenteritis (PGE), which is a parasitic disease of major socioeconomic importance in farming communities worldwide [2]. In small ruminants, the GINs of greatest importance belong to the superfamily Trichostrongyloidea, and the most important genera are *Haemonchus*, *Teladorsagia*, and *Trichostrongylus* [3]. To a lesser extent, members of the genera *Nematodirus* and *Cooperia* and those in the superfamily Strongyloidea (genera *Chabertia* and *Oesophagostomum*) also commonly parasitize sheep and goats. These latter species cause disease only in exceptional circumstances but may also contribute to PGE. Nevertheless, the manifestation and severity of the disease are affected by a broad range of host–parasite-related factors such as host immunity and nutrition, as well as parasitic species richness and intensity of infection [4]. In general, more severe clinical manifestations, typically diarrhoea and weight loss, develop with heavier nematode burden, and anaemia associated with the blood-sucking habit of *Haemonchus* makes it a highly pathogenic parasite. Therefore, even regular observation of animals may not reveal the extent of parasite burden until PGE develops into a severe clinical form [2].

GIN infections in ruminant livestock are typically controlled by anthelmintic drugs [5]. However, the excessive and, in some cases, incorrect use of these chemicals has escalated over the past few decades into widespread dissemination of resistant nematode populations among livestock herds and even to a high prevalence of multi-drug resistance to anthelmintics in some regions of the world [6, 7]. The situation with high GIN burden in small ruminants, together with increasing anthelmintic drug resistance, leads us to focus our efforts towards integrated control with precisely justified and species-dependent drug use, whose cornerstone is fast and reliable diagnostics [8, 9]. There is no worldwide consensus on the best sampling strategy and diagnostic procedure for GIN screening in sheep [10], but traditional coprological methods are regarded as limited and unreliable [11, 12]. From this point of view, the precise diagnostics remain challenging. The limitation of conventional faecal flotation techniques is in their inability to reliably distinguish individual strongyle genera, given the fact that the morphology of the eggs is extremely similar between species (with the exception of *Nematodirus* spp.) [13]. For identification to genus level and beyond, time-consuming larval culture (LC) is routinely used, enabling the development of extracted eggs to third-stage larvae (L_3_), which can then be microscopically differentiated based on both their morphological and morphometric characters [14]. LC has limitations related not only to different requirements for hatching and larval development of individual nematode species but also on the experience of the investigator and ambiguous taxonomic distinction [12, 15].

Polymerase chain reaction (PCR)-based methods have shown high sensitivity and specificity for the detection of parasite DNA, yet their use for in vivo diagnostics of GIN infections directly from faeces has so far been limited to only three studies by Höglund et al. [10], Sweeny et al. [16], and Roeber et al. [17]. The focus of GIN diagnostics using molecular methods is still mainly related to extracted eggs [11–13, 18–22] and/or cultivated larvae [13, 14, 22, 23]. Faecal DNA extraction is still believed to be unsuitable for usage in most subsequent PCR amplifications due to the presence of faecal inhibitors [24]. However, developments in DNA extraction procedures and application of inhibitor removal technologies have made such DNA ideal for subsequent PCR-based analyses, especially real-time PCR. This has made this technology a powerful alternative or additional tool to coproscopy [25].

In the present study, we designed two multiplex real-time PCR assays, which included internal amplification control, targeting the internal transcribed spacers 1, 2 (*ITS1/2*), small subunit rRNA (*SSU rRNA*), and cytochrome c oxidase subunit I (*COI*) genetic markers to diagnose naturally acquired GIN infections in sheep. We developed six specific detection systems and describe their use for the detection of genomic DNA of the nematode species *Haemonchus contortus*, *Teladorsagia circumcincta*, *Trichostrongylus colubriformis*, *Nematodirus battus*, *Ashworthius sidemi*, and *Chabertia ovina*, respectively. We further tested the functionality of both multiplex assays on 44 individual faecal samples from sheep and compared the results of a semi-quantitative evaluation of infection intensity to the egg enumerations generated by a conventional faecal flotation technique. The ability to reliably identify all targeted GINs present within faeces and rank them according to their numerical contribution in a single reaction represents a major advantage over conventional coprological techniques. This approach has clear, practical value, is cost-effective and efficient, and hence is widely applicable in the control of parasites in ruminant livestock and epidemiological studies.

## Methods

### Biological material

The optimization experiments were carried out with genomic DNA (gDNA) extracted from adult nematodes stored in 70% ethanol, which were recovered from the gastrointestinal tracts of ruminants using standard *post mortem* examination. The host animals came from various areas of the Czech Republic, and sampling was conducted in 2017–2019. The recovered nematodes were identified based on their dominant distinguishing morphological/morphometric characters using an Olympus BX51 light microscope, and measurement of their dominant morphological characteristics was carried out by QuickPHOTO MICRO 3.0 software (PROMICRA, Prague, Czech Republic). Adult nematodes were determined as *H. contortus*, *T. circumcincta*, *T. colubriformis*, *N. battus*, *A. sidemi*, *Ch. ovina*, *Cooperia curticei*, *C. pectinata*, and *Oesophagostomum columbianum*. One lamb experimentally infected with *H. contortus* served as a source of eggs for accurate assessment of nematode eggs in faeces.

In total, 44 individual faecal samples for functional verification of the multiplex assays were collected rectally on three farms (F1, F2, and F3) during autumn of 2019 in the Czech Republic. Approximately 5 g of faeces from the rectum of approximately 10% of the animals in the flock was collected and stored in plastic zipper bags and, after transportation, frozen at −80 °C until further processing for molecular analyses. At the same time, another batch of 5 g of faeces originating from the same animals was collected for immediate quantitative coprological examination using the concentration McMaster technique with minimum diagnostic sensitivity of 20 strongyle eggs per gram of faeces (EPG) [26]. Besides strongylid-type egg enumeration, the presence of *Nematodirus* spp. eggs (includes all species except *N. battus*, which is distinguished separately) was also recorded for each sample.

In addition to coprological examination, about 1 kg of pooled faecal sample from the whole flock was collected at each farm and incubated under standard conditions to obtain infective L_3_ larvae. Faecal pellets were mixed in a beaker with vermiculite chips, manually homogenized and then kept in moist conditions in an incubator at 28 °C for 1 week. The developed L_3_s were recovered using Baermannization and identified based on their morphological/morphometric features to the genus level according to morphological keys in van Wyk et al. [15] and van Wyk and Mayhew [27].

### DNA extraction

The gDNA of adult nematodes, which served as reference template DNA during the optimization experiments, was extracted from a single individual of each given nematode species using previously published protocols [28, 29] and based on overnight incubation at 55 °C in 50 μl of extraction buffer (100 mM Tris–HCl, 10 mM EDTA, 100 mM NaCl, 1% SDS, 1.5 mM dithiothreitol) containing 0.06 mg proteinase K, followed by alcohol precipitation. Extracted gDNA was stored at −20 °C until processed.

Faecal samples were thawed before the extraction, and each zipper bag containing sample was thoroughly manually homogenized. Out of the homogenized faeces, 1 g was taken for further processing and extraction using the Quick-DNA Fecal/Soil Microbe MiniPrep kit (Zymo Research, Irvine, CA, USA). Each sample was placed in a sampling container and dissolved by shaking in 800 μl of BashingBead Buffer mixed with 3200 μl of PBS buffer. A negative isolation control (NIC), i.e., a tube without faeces, starting from this step through the whole extraction procedure together with the samples, was included in every extraction procedure. Then, 1200 μl of the sample solution was transferred into a ZR BashingBead Lysis Tube and mechanically lysed, ensuring the disruption of eggs and further homogenization of other debris, in a Retsch MM200 mixer mill (RETSCH, Haan, Germany) for 10 min. Then, lysis tubes were centrifuged, and the supernatant transferred to a Zymo-Spin III-F Filter; the further procedure faithfully followed the kit manufacturer’s protocol. After elution, DNA samples were diluted tenfold and stored at −20 °C until use.

Concentration, yield, and purity of all extracted DNA were measured by a NanoDrop 8000 Spectrophotometer (Thermo Fisher Scientific).

### Detection systems

Target gene sequences of nematode species of interest were downloaded from the NCBI GenBank database and aligned to find conserved genus-specific regions suitable for oligonucleotide hybridization. Suitable sequence regions were chosen manually and tested in the OligoAnalyzer 3.1 online tool (Integrated DNA Technologies, Coralville, IA, USA) for melting temperatures and formation of secondary structures. The specificity of each primer probe set was evaluated in silico in Nucleotide BLAST and considered during the design process, following the guidelines by Rodríguez et al. [30]. Based on accessible sequences and their reaction properties, six detection systems were designed: *Haemonchus* (*ITS2*; system was designed to cover species *H. contortus* and *H. placei*), *Teladorsagia* (*ITS2*; covering *T. circumcincta* and *T. trifurcata*), *Trichostrongylus* (*ITS1*; covering *T. colubriformis*, *T. vitrinus*, and *T. rugatus*), *Nematodirus* (*SSU rRNA*; designed for *N. battus*), *Ashworthius* (*ITS1*; designed for *A. sidemi*), and *Chabertia* (*COI*; designed for *Ch. ovina*). Dual-labeled hydrolysis probes carried a reporter dye (FAM, HEX, TxRd, or Cy5) at the 5′ end and a compatible dark quencher at the 3′ end. Primers and probes (Additional file 1: Table S1) purified by high-performance liquid chromatography (HPLC) were purchased from Sigma-Aldrich.

The experimental evidence of the unique specificity for each target was validated for each designed detection system by testing homologous and heterologous combinations of the specific set of primers and probes with equimolar amounts of template gDNA (1 ng) extracted from adult nematodes (*H. contortus*, *T. circumcincta*, *T. colubriformis*, *N. battus*, *A. sidemi*, and *Ch. ovina*), as well as other closely related species (*C. curticei*, *C. pectinata*, and *O. columbianum*) commonly parasitizing small ruminants. This evidence was then further verified in multiplex form of the reaction mixture (Table 1).

**Table 1:** Specificity of multiplex assays evaluated with gDNA of adult nematodes, expressed by Cq values

DNA template	Detection system					
	Multiplex1			Multiplex2		
	*Haemonchus*	*Teladorsagia*	*Trichostrongylus*	*Nematodirus*	*Ashworthius*	*Chabertia*
*Haemonchus contortus*	23.51	nd	nd	nd	nd	nd
*Teladorsagia circumcincta*	nd	25.54	nd	34.73^a^	nd	nd
*Trichostrongylus colubriformis*	nd	nd	24.05	nd	nd	nd
*Nematodirus battus*	nd	nd	nd	26.23	nd	nd
*Ashworthius sidemi*	nd	nd	nd	nd	27.07	nd
*Chabertia ovina*	nd	nd	nd	nd	nd	25.18
*Cooperia curticei*	nd	nd	nd	nd	nd	nd
*Cooperia pectinata*	nd	nd	nd	nd	nd	nd
*Oesophagostomum columbianum*	nd	nd	nd	nd	nd	nd

^a^Cq value that corresponds to nonspecific amplification. *Abbreviation*: nd, no DNA was amplified

Sensitivity was first tested in separate reactions for each system and was further verified in multiplex and on mixed template DNA. Serial tenfold dilutions (10 ng to 10 fg per μl) of stock solutions of gDNA of adult nematodes were used for the tests; similarly, in the case of an experiment with the DNA mixtures, template DNA of three targets was pooled (with respect to a certain multiplex assay) and diluted in the same manner. To determine whether a non-competitive environmental DNA could interfere with the assay, 10 µg of an excess DNA from fish sperm (SERVA, Heidelberg, Germany) was added to the samples prior to testing.

### Real-time PCR standard curve and internal control

A plasmid DNA construct serving as a positive control in the reaction was prepared based on the *ITS2* sequence of *H. contortus.* The same amplified sequence as targeted in the relevant multiplex assay by specific primers to *H. contortus* was cloned via CloneJET (Thermo Fisher Scientific, Waltham, MA, USA) into vector pJET1.2/blunt and transformed into DH10B-competent *E. coli* cells. The plasmid was purified with the NucleoBond Xtra kit (Macherey–Nagel, Düren, Germany), and its DNA concentration determined by NanoDrop. The plasmid construct was sequenced to confirm the proper insertion of the intended sequence. The copy number was calculated using the formula: copies/μl = X ng * 6.022 × 10^23^ molecules/mole / [(*N* * 660 g/mole) * 1 × 10^9^ ng/g], where X is the concentration of the plasmid [ng/μl] and *N* is the length of the dsDNA [plasmid with insert]. The calculated copy number was used for preparation of the plasmid stock solution 1 × 10^10^ (34.1 ng/µl) in 50 μg/ml of carrier DNA from fish sperm, and the standard curve was generated by serial dilutions of the plasmid stock to give 5 × 10^7^, 1 × 10^7^, 5 × 10^6^, 1 × 10^6^, 5 × 10^5^, 1 × 10^5^, 5 × 10^4^, and 1 × 10^4^ copies per microlitre. This series was analysed in duplicate by real-time PCR using a Multiplex1 (M1) assay in three replications (three separate plates) to test the efficacy and reproducibility of the reaction. To relate the technical calibration curves of plasmid control to the number of eggs in sheep faeces, we spiked 5 g of parasite-naïve (trichostrongyle egg-free) sheep faeces with a given number of *H. contortus* eggs. Two independently diluted faecal concentration series ranging from 7500 to 1 EPG were processed following the DNA extraction for faecal samples described above. We analysed the samples in duplicates and in three replications using M1 assay simultaneously with plasmid control.

The sequence of the internal amplification control (IAC) was created by Mikel et al. [31] from ancient mitochondrial DNA sequences of two extinct species and synthesized de novo (Sigma-Aldrich, St. Louis, MO, USA). This non-competitive synthetic sequence was cloned into a plasmid, purified and diluted using the same protocol as with *H. contortus* plasmid control. The reaction premix of both multiplex assays then contained IAC specific primers, hydrolysis probe (Additional file 1: Table S1), and plasmid serving as its template to differentiate between truly negative and false-negative (inhibited) samples [32].

### Real-time PCR reactions and data analysis

All experiments were based on two four-plex real-time PCR assays using TaqMan technology, where M1 consisted of detection systems *Haemonchus*, *Teladorsagia* and *Trichostrongylus*, and Multiplex2 (M2) combined detection systems *Nematodirus*, *Ashworthius* and *Chabertia;* the detection system for IAC was identical in both multiplex assays. The conditions of multiplex assays were altered systematically until the optimal primer and probe concentrations, concentration of IAC and cycling conditions were determined. The composition of optimized reaction mixture in a final volume of 20 μl contained: 1X Luna Universal Probe qPCR Master Mix (New England Biolabs, Ipswich, MA, USA), 250 nM of each of the eight primers, 100 nM of FAM, TxRd and Cy5 probes and 200 nM of HEX probe, 0.4 U of Antarctic Thermolabile UDG (New England Biolabs), 1 × 10^4^ copies of IAC plasmid and 5 μl template DNA. All samples were run in duplicates on a CFX96 Touch Real-Time PCR Detection System (Bio-Rad Laboratories, Hercules, CA, USA) using 96-well PCR plates under the following conditions: incubation step at 25 °C for 10 min (carryover prevention), initial denaturation at 95 °C for 2 min followed by 40 cycles of 95 °C for 15 s and 57 °C for 45 s, and a final cooling step of 40 °C for 30 s.

Plasmid standard, no-template control (NTC), and NICs (in the case of real sample analysis) were included on each analysed plate, while IAC was incorporated in every sample, inclusive of controls. The raw data were analysed using the Bio-Rad CFX Manager 3.0 (Bio-Rad Laboratories). The quantification cycle (Cq) values of the samples were read after adjustment of the relative fluorescence units (RFU) threshold line to 140 for FAM, 120 for HEX and 210 for TxRd channel. The sample was considered to be positive only when both replicates provided a positive result and conversely, the sample was considered to be negative only when both replicates gave a negative result, but the IAC gave a positive signal; if this condition was not met, the real-time PCR and/or the DNA extraction was repeated.

## Results

### Optimizations

In the specificity tests, no nematode DNAs other than those targeted were amplified and, in all samples, there was a clear signal for the IAC. The only exception was found in the detection system for *Nematodirus* where 1 ng of *T. circumcincta* DNA was non-specifically amplified with Cq 34.73 (Table 1). However, this was not observed in the testing of real samples, even though fluorescent signal for *Teladorsagia* has been reported frequently.

The limit of detection (LOD) was the same or better when the mixed samples were run in multiplex mode than when single template DNA was run in either in singleplex and multiplex (see Additional file 2: Tables S2–S3). The LOD of detection systems in multiplex assays was determined to be at least 500 fg of target DNA with the exception of the *Ashworthius* system, where the LOD was 5 pg (Table 2). An addition of non-competitive excess DNA from fish sperm had no impact on assay performance.

**Table 2:** Evaluation of sensitivity and limit of detection of detection systems in multiplex assays expressed by Cq values

Mixed template DNA	Detection system					
	Multiplex1			Multiplex2		
	*Haemonchus*	*Teladorsagia*	*Trichostrongylus*	*Nematodirus*	*Ashworthius*	*Chabertia*
50 ng	16.54	17.29	17.54	18.79	18.82	16.23
5 ng	19.82	20.50	21.05	22.34	22.26	19.80
500 pg	23.70	24.32	24.67	26.31	25.86	23.42
50 pg	27.35	27.96	28.16	30.01	29.48	26.91
5 pg	31.10	32.18	31.60	34.06	34.61	30.11
500 fg	34.74	35.90	35.23	38.41	nd	33.54
50 fg	nd	nd	nd	nd	nd	nd

The sensitivity was assessed with equimolar mixtures of gDNAs of adult nematodes (in particular, DNA template for Multiplex1 consisted of *H. contortus*, *T. circumcincta*, and *T. colubriformis*, and Multiplex2 of *N. battus*, *A. sidemi*, and *Ch. ovina*). *Abbreviation:* nd, no DNA was detected

### Efficacy and reproducibility

**Fig. 1:**
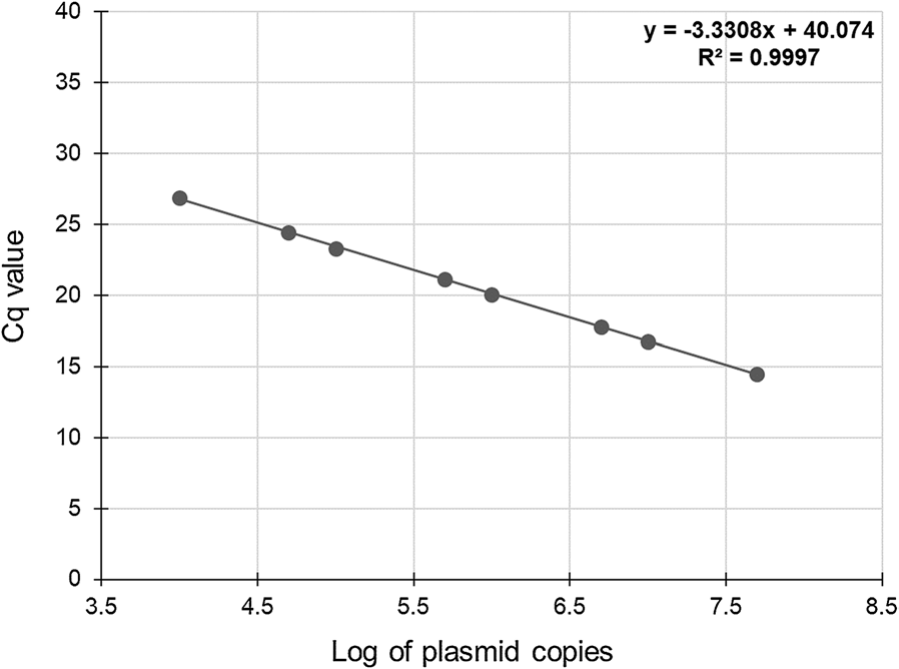
A calibration curve of *Haemonchus contortus ITS2* plasmid standard. The data points are the median of three replicates. The equation and *R*^*2*^ linearity value resulting from the linear regression analysis are shown in the graph

Based on the median values of *H. contortus ITS2* plasmid standard calibration curve (Fig. 1), the PCR efficiency was established to be 99.6%, correlation coefficient 0.9997, slope −3.3308 and *y* intercept 40.074. The standard deviation (SD) of the Cq values within the plasmid DNA replicates between plates was < 0.11 and the coefficient of variation (CV) < 0.8% (Table 3).

**Table 3:** Repeatability and reproducibility of multiplex real-time PCR reactions

Plasmid copies per µl	Median ± SD ^a^	CV (%)
5 × 10^7^	14.49 ± 0.11	0.8
1 × 10^7^	16.74 ± 0.04	0.2
5 × 10^6^	17.78 ± 0.05	0.3
1 × 10^6^	20.02 ± 0.10	0.5
5 × 10^5^	21.12 ± 0.05	0.2
1 × 10^5^	23.30 ± 0.10	0.4
5 × 10^4^	24.44 ± 0.06	0.3
1 × 10^4^	26.84 ± 0.08	0.3

^a^ Median ± standard deviation (SD) and coefficient of variation (CV) of Cq values of three repetitions of *H. contortus* *ITS2* plasmid standard

### Semi-quantitative data evaluation

**Fig. 2:**
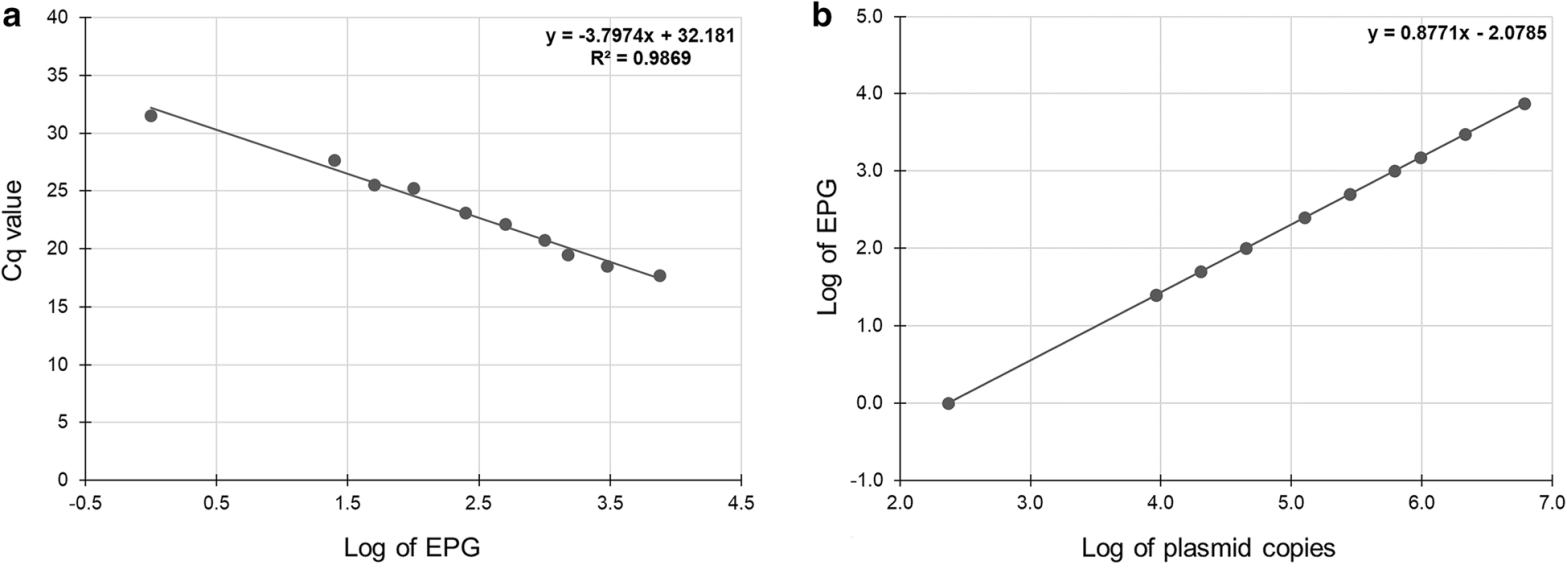
A semi-quantitative estimation of the number of eggs in unknown samples. **a** A calibration curve of faecal concentration series ranging from 7500 to 1 EPG. The data points are the median of three replicates of two independently diluted series. The equation and *R*^2^ linearity value resulting from the linear regression analysis are shown on the graph. **b** The formula serves to convert plasmid copy numbers to EPG and was created by assignment of plasmid standard copy numbers to corresponding EPG counts correlated based on faecal concentration series

Based on median values of faecal series calibration curve (Fig. 2a) the PCR efficiency was established to be 83.4%, correlation coefficient 0.9869, slope −3.7974 and *y* intercept 32.181. The SD of the replicates between plates was < 0.59 and CV < 2.1%.

The Cq values of faecal concentration series ranging from 7500 to 1 EPG were correlated with the corresponding values of *H. contortus* *ITS2* plasmid standard copies, enabling a semi-quantitative estimation (Fig. 2b) of the number of eggs in unknown samples.

Since not all GIN detection systems use the same target marker gene, "correction factors" were calculated to overcome their different representation in the respective genome (an issue for all *ITS* sequences [13]). Equimolar mixed gDNAs of adult nematodes were tested repeatedly, identifying slight biases in performance between the different molecular targets. Since these biases obtained by comparing Cq values of samples of the same DNA concentration were similar for individual targets in different nematode isolates, replicates, and plate replications, the correction factors were established as follows, taking *H. contortus* as the reference value (1): *T. circumcincta* (0.893; 95% confidence interval [CI]: 0.853–0.934), *T. colubriformis* (0.966; 95% CI: 0.943–0.989), *N. battus* (0.910; 95% CI: 0.889–0.930), *A. sidemi* (0.879; 95% CI: 0.861–0.897), and *Ch. ovina* (0.995; 95% CI: 0.967–1.022).

To enable evaluation, the Cq value of each detected target sequence in the analysed sample was firstly assigned to plasmid copy number counting with the calibration curve of plasmid control on the plate, sample dilution, and respective correction factor. The plasmid copy number was then used to estimate the EPG value of the target, based on the formula for semi-quantitative evaluation (Fig. 2b). EPG values of individual targets of the sample were summed to obtain the estimation of overall infection intensity (total EPG), whereas the limit values for interpretation were set to < 100 EPG in low infection, 100–1000 EPG medium infection, and > 1000 EPG high infection; the same cut-off values were used for evaluation of faecal egg count (FEC) results.

It was found that 1 EPG corresponded to 234 copies of plasmid standard, which was considered a minimum limit for a positive detection in the faecal samples.

### Functional verification of the multiplex assays

In this study, a total of 44 individual faecal samples from three sheep farms in the Czech Republic were examined using the faecal flotation technique and tested by two four-plex real-time PCR assays to verify the functionality of the designed assays and to evaluate the validity of the proposed semi-quantitative evaluation. Strongylid-type eggs were detected by the concentration McMaster technique in 40 (90.9%) out of the 44 faecal samples collected on three sheep farms (Table 4), with the exception of *Nematodirus* spp., which was found/identified in only six samples (13.6%). In contrast, strongylid DNA was detected by the multiplex real-time PCR in 43 (97.7%) samples (Table 4); the highest percentages of test-positive samples were related to *Haemonchus* (88.6%), followed by *Teladorsagia* (81.8%), *Chabertia* (72.7%) and, to a lesser extent, *Trichostrongylus* (61.4%), whereas both *N. battus* and *A. sidemi* DNA were absent.

**Table 4:** Functional verification of the multiplex assays based on analysis of 44 faecal samples

No. of samples tested	No. of samples test-positive by the faecal flotation technique			No. of samples test-positive by multiplex real-time PCR					
	Total	Strongylid-type eggs	*Nematodirus* spp.	Total	*Haemonchus*	*Teladorsagia*	*Trichostrongylus*	*Chabertia*	Mixed infections
44 (100.0%)	40 (90.9%)	40 (90.9%)	6^a^ (13.6%)	43 (97.7%)	39 (88.6%)	36 (81.8%)	27 (61.4%)	32 (72.7%)	43/44 (97.7%)

Each sample was subjected to quantitative coprological examination using the concentration McMaster technique for enumeration of strongylid-type eggs and eggs of *Nematodirus* spp. (includes all species except *N. battus*). After DNA extraction, multiplex real-time PCR analysis was performed, targeting within two assays *Haemonchus* (*H. contortus, H. placei*), *Teladorsagia* (*T. circumcincta, T. trifurcata*), *Trichostrongylus* (*T. colubriformis, T. vitrinus*, and *T. rugatus*), and *Chabertia* (*Ch. ovina*). *Nematodirus* (*N. battus*) and *Ashworthius* (*A. sidemi*) DNA was not detected

^a^ *N. battus* was described in one sample

In addition, total EPG calculated from Cq value and the relative proportions of the different nematodes in each sample were compared with the FEC from the faecal samples of the same individuals determined by the concentration McMaster technique (Additional file 3: Table S4). There was fair agreement in the evaluation of the infection severity between results obtained by these two techniques/methods when based on total EPG (weighted kappa 0.315 ± 0.107) and moderate agreement when based on the results of the most pathogenic genus, *Haemonchus* (weighted kappa 0.478 ± 0.109). Only one sample (No. 21) tested negative for all screened targets by both techniques, although having positive IAC indicated that faecal inhibitors were not responsible for the negative results in multiplex real-time PCR. Three samples (No. 25, 31 and 34) tested negative by coprological examination but positive in multiplex assay, whereas samples No. 31 and 34 were evaluated as severe infections. Mixed infections were observed in 100.0% of positively tested samples with at least two trichostrongyle representatives detected.

To further compare coproscopy to molecular approach, LC prepared from the pooled flock samples were included. GIN composition on farms based on morphological identification of L_3_ was evaluated as follows: at F1 were differentiated in 73.0% as *Haemonchus*, 10.0% *Teladorsagia*, 8.0% *Trichostrongylus*, and 9.0% *Chabertia*; F2 was in 41.0% *Haemonchus*, 22.0% *Teladorsagia*, 17.0% *Trichostrongylus*, and 20.0% *Chabertia*; and finally, at F3 was identified in 48.0% *Haemonchus*, 18.0% *Teladorsagia*, and 34.0% *Trichostrongylus*.

## Discussion

PGE in ruminants is usually caused by a mixture of GINs occurring simultaneously [3, 33]. Therefore, the potential implications for studying these parasites (population biology, epidemiology, treatment efficacy, etc.) offered by quantitative and multiplex PCR assays seem highly valuable. Apart from the order of magnitude higher price of molecular methods compared to traditional coproscopy, the advantages are manifold. The most valuable is time savings and precision of identification while maintaining simultaneous detection of multiple parasites present in a sample (use of three different dyes for the six targets means that only one third of the number of reactions must be performed to diagnose all six GINs), high-throughput capacity, wide dynamic range, and avoidance of inaccuracies related to end-point analysis. High-throughput sequencing devices are being increasingly applied to the challenge of GIN identification [34] and complex nematode community studies [35–37]. This ‘nemabiome’-type technology is still relatively expensive and requires expertise and complicated bioinformatic analysis, so is still the preserve of specialist laboratories. In contrast, real-time PCR technology is very widespread today and has become common equipment of larger laboratories, allowing diagnostic procedures based on these platforms to be used relatively routinely. This fact, together with time and laboratory simplicity, ensures immediate results without bioinformatic pipelines, bringing our present multiplex real-time PCR assays for specific detection and semi-quantitative evaluation of the most prevalent GIN infections directly from faeces of domestic livestock closer to the wider scientific community, and in the future could represent a golden mean in terms of costs and outputs.

Roeber et al. [17] developed an automated multiplexed-tandem PCR (MT-PCR), with properties very similar to our approach, with the exception that the individual species real-time components are carried out in parallel, in separate reactions, rather than simultaneously. The platform, targeting *ITS2* regions of six GINs (*Haemonchus* spp., *T. circumcincta*, *Trichostrongylus* spp., *Oesophagostomum* spp., *Ch. ovina*, and *C. curticei*), was validated for European applications [22] and showed promise for routine use in practice. Although they tested direct extraction of DNA out of 0.25 g of faeces using the PowerSoil DNA purification kit (MoBio) as one of the possible methods in the MT-PCR study [17], the results showed decreased detection sensitivity, which led to the decision for the continued use of previously extracted eggs/L_3_ larvae only. In comparison, we see the advantage of our approach in needing no additional equipment, such as handling robot devices, and in the usage of faecal samples directly for DNA extraction. The utilization of pre-extraction steps focused on proper homogenization and sample dissolution and the usage of specialized kit with unique filtration technologies effectively removing PCR inhibitors improved the extraction process and overcame this issue in the case of our study. These provisions ensured higher sample input, increased diagnostic sensitivity, and reduced the overall turnaround time. This was demonstrated by detecting samples false-negative by FEC (possibly in a prepatent stage of infection) and by the absence of PCR inhibition evidenced by IAC amplification.

The parasites for which the multiplex real-time PCR assays are described in this study represent, apart from *A. sidemi*, the most important GIN of sheep. The oligonucleotides designed for this study show sequence identity with the currently available NCBI GenBank database gene sequences of individual targets (data not shown). Therefore, even though we did not investigate the specific detection of further targeted species of the same genera (namely *H. placei*, *T. trifurcata*, *T. vitrinus*, and *T. rugatus*) due to the unavailability of DNA material, it can be assumed that the respective probes will detect even these closely related species for which the present detection systems were intended [14].

*Ashworthius* detection was included in the presented multiplex assay for the following reasons. This hematophagous abomasal nematode is phylogenetically related and morphologically/morphometrically almost indistinguishable in immature stages from *H. contortus*, and poses a threat of becoming one of the most widespread pathogenic GINs of autochthonous European ruminants [29]. This invasive parasite, originally endemic in Asian deer, was probably introduced to Europe by sika deer in the late nineteenth and early twentieth century, and since then it has successfully spread among new hosts (such as red deer, roe deer, fallow deer, or moose) and is highly pathogenic in some, such as European bison [38]. The susceptibility of domestic sheep to ashworthiosis has so far only been confirmed experimentally [39], whereas the first cases of natural infection in cattle were recorded in Poland by Moskwa et al. [40]. Thus, horizontal transmission of this parasite from wild ruminants to domestic livestock is probably just a matter of time in all regions where these hosts share the same pastures. Therefore, having access to a reliable diagnostic tool that can detect *A. sidemi* in livestock was the main purpose of including this parasite in our multiplex assay.

Detection systems for members in the genus *Nematodirus* are typically not included in similar studies devoted to strongyle nematodes of livestock due to their distinguishability based on the eggs, which are much larger and contain distinctive dark cells. However, *N. battus* is an especially important pathogen in temperate climates, causing significant health problems in grazing lambs in spring. For this reason, we decided to include this species in the multiplex analysis of DNA extracted directly from sheep faeces, which could be used as an alternative tool to coproscopy. During the assay specificity tests, we noticed a weaker non-specific amplification of *T. circumcincta* DNA (Table 1) with the *Nematodirus* system. However, this phenomenon was not recorded during tests on faecal samples, even in those with a high EPG in the *Teladorsagia* system (Additional file 3: Table S4). This anomaly could either be caused by the presence of *Teladorsagia* species other than *T. circumcincta*, to which the *N. battus* probe did not bind due to sequence variations, or more likely due to the presence of an excess amount of non-specific DNAs in a complex sample, such as faeces, that might block non-specific hybridization during the PCR reaction the same way as sheared DNAs [32, 41, 42]; such enhancement of specificity has previously been demonstrated by the usage of competing primers [43] or the presence of tRNA [44]. In the present study, *Nematodirus* spp. were detected only in six faecal samples out of 44 examined by the concentration McMaster technique (Table 4). In five of these samples (No. 32, 41–44) the *Nematodirus* spp. eggs were identified, while in one (No. 8), *N. battus* was also present but only judged on a single egg. Sample No. 8 was re-extracted and tested repeatedly by multiplex real-time PCR but always with negative results. This could be as a result of the misidentification of *Nematodirus* species based on a single egg (since species other than *N. battus* would not be detected by our assay, e.g., *N. filicollis*, *N. helvetianus*), or sampling error (no eggs were present in the faecal sample used for molecular analysis), or because the amount of DNA was below the LOD for this system. The use and suitability of this detection system need to be further evaluated during a broader study on field samples from sheep flocks.

The LOD determined by assay sensitivity tests on adult nematodes gDNAs (Table 2) cover the range of 0.5–5 pg, which equates to a proportion of gDNA extracted from a single egg [11]. These results are consistent with previous studies based on other singleplex [11, 21] and multiplex real-time PCR assays [25]. To refine the data interpretation for faecal samples, the limit of 1 EPG corresponding to 234 plasmid copies was established. In the present study, this corresponded to Cq 35.51 in *Haemonchus*, Cq 39.75 in *Teladorsagia*, Cq 36.77 in *Trichostrongylus*, Cq 39.03 in *Nematodirus*, Cq 40.00 in *Ashworthius*, and Cq 35.70 in *Chabertia* detection systems, which were all derived from the median values of a plasmid standard.

Based on IAC, which was amplified in all samples, there was no evidence of inhibition in the PCR following the column extraction of gDNA directly from faeces. Due to recent improvements in commercial faecal extraction kits towards purification of the sample suitable for downstream molecular-based applications, faecal inhibitors themselves are no longer the biggest concern. Harmon et al. [18] pointed out that the factor causing the majority of the variance in Cq values between samples containing the same numbers of eggs is partly attributable to the DNA extraction procedure, when the variation seen from amplified egg DNA exceeded that obtained with plasmids. They also suggested that these variances are corrected in multiplex reactions where intra-sample comparisons are made for each trichostrongyle target [18]. Similarly to Avramenko et al. [36], we calculated genus-specific correction factors to eliminate any sequence representation bias (which could be given for example by the different target sequence features or their copy number in respective genomes). Still, the limit values 0.879–1.0 of their range points to the minimal effect of these parameters on resulting values.

Bott et al. [11] used a microscopic McMaster approach combined with molecular real-time PCR followed by melting curve analysis optimized for extracted eggs, and suggested that such a platform can be used for the semi-quantitation of target DNAs and infection intensity evaluation based on Cq values of individual targets. The reason for this consideration was that semi-quantitative evaluation together with knowledge of species/genus composition is more valuable than absolute quantification, given that the intensity of nematode infection does not have to reflect the number of eggs excreted per gram of faeces; the exception to this are nematodes with high biotic potential (e.g., *H. contortus* and Oesophagostominae) [11]. Based on this knowledge and experience, we conclude that a semi-quantitative enumeration of different GIN eggs in faeces based just on *ITS2* *H. contortus* plasmid control is feasible by our multiplex real-time PCR assays. The results of individual faecal samples (Additional file 3: Table S4) and their comparison to FEC suggests, with the moderate agreement in the evaluation of the infection severity, that genus *Haemonchus* is confirmed as the most pathogenic GIN in sheep [45], being widespread and highly prolific, the trigger point for diagnostic evaluation and the selection of follow-up measures, at least under European conditions. Knowledge of the representation of other species in the sample adds further diagnostic value and may influence the choice of subsequent control and treatment procedures; these may then differ from those determined just on the basis of FEC results. Testing of the 44 individual faecal samples revealed three samples where the presence of nematodes remained undetected by the coproscopy, but this negativity turned out to be false by multiplex real-time PCR. However, this was not noted the other way around, i.e., there were no samples positive in FEC which would test negative by molecular analysis. This indicates that the molecular approach is more sensitive for the detection of eggs directly from faeces than the concentration McMaster technique, where one egg equates to 20 EPG. These observations are basically in agreement with earlier results by Sweeny et al. [16]. Nematode composition based on LC proportionally agreed with that resulting from multiplex assays; however, a slight bias can be observed in the example of F1, where *Trichostrongylus* DNA was not detected but microscopically determined at 8.0% according to LC results. Since *Teladorsagia* was the second most abundant genus on this farm, this indicates a confusion of *Trichostrongylus* and *Teladorsagia* larvae during microscopical identification of L_3_. Usually these two genera are grouped together even with an experienced observer [27]. The high sensitivity of the present multiplex real-time PCR and the use of an individual animal testing approach, preventing bias of the result due to animals with aggregated parasites, are the required features for reliable GIN diagnostics.

Although emphasis must be placed on proper homogenization and mechanical cell disruption of the faeces, there are several advantages of using multiplex real-time PCR assays compared to LC. The most noticeable advantage is the significant shortening in number of steps included in the procedure, resulting in an overall reduction in procedure duration from 7 days on cultured L_3_ [46], 2 days with L_1_ [14], to only a few hours in the case of the present multiplex assays. Detection of specific DNA also enables GIN diagnostics on frozen faecal samples, where the vast majority of eggs are distorted or are otherwise damaged, which could result in decreased ability to float.

Another indisputable but little known and underestimated advantage of using biological samples directly is the presence of parasite cell-free DNA (cfDNA). CfDNA is released during disintegration of different life stages (cellular apoptosis/necrosis) and active secretion from cells, and its presence (and other parasite components) within the faeces cannot be identified by microscopy [47]. The detection of cfDNA is being increasingly applied as an efficient biomarker for the accurate diagnosis of parasites occupying tissues and the circulatory system. The presence of extracellular cfDNA helps to overcome problems connected with intermittent egg shedding, and thus greatly increases the sensitivity and accuracy of the semi-quantitative diagnostic assay. Its presence can also reveal the prepatent stage or hypobiotic larvae (arrested larval development) [11], since cfDNA is released by the larvae present in the wall of the gastrointestinal tract. The theory of cfDNA and its potential to increase assay accuracy might be supported by individual faecal sample results that tested negative by microscopical FEC; in the case of sample No. 25, we assume that a low intensity of infection detected by multiplex assay (total EPG = 44) could adversely affect faecal flotation, which may be subject to a dilution error, but this was presumably not the case in the other two samples. Samples No. 31 and 34 tested negative by faecal flotation but highly positive by multiplex real-time PCR [25], with rankings of severe infections. In both samples, high levels of *Haemonchus*, *Teladorsagia*, and *Trichostrongylus* were detected. A possible explanation for these results could be a prepatent period of infection with a high representation of developing larval stages. Although less likely scenarios such as some form of hypobiosis or sampling error given, for example, by intermittent egg shedding, cannot be ruled out; the faeces for both tests were collected at the same time of the day, and we reckon that in such a heavy infection at least some eggs would be present anyway.

No molecular approach for specific DNA detection of GINs in sheep using fresh faeces has yet been implemented in standard diagnostic practice. In our opinion, the two multiplex real-time PCR assays presented in this study may enable reliable evaluation of GIN genus/species richness within European sheep flocks and thus improve diagnosis of GIN infections in ruminant livestock. This preliminary evidence of the assays’ ability to rapidly identify and rank nematodes according to their numerical contribution to observed FEC in mixed infections may also suggest the potential to become a powerful alternative or practical adjunct to conventional faecal egg count reduction tests (FECRT), to enable the rapid inference of which strongyle species or genera are susceptible or resistant to particular anthelmintic drugs [12, 16]. However, further evaluation and refinements of these assays need to be carried out during further testing on real samples to increase their precision; then they could usefully supplement or replace the conventional coprological techniques for the purpose of routine diagnosis.

## Conclusions

Two multiplex real-time PCR assays designed in this study, containing among other things an internal amplification control to avoid false-negative results, targeting the main GINs of sheep were successfully tested for detection of *H. contortus*, *T. circumcincta*, *T. colubriformis*, *N. battus*, *Ch. ovina*, and, atypically, *A. sidemi* as an invasive threat to sheep flocks. These assays were optimized to be performed using DNA extracted directly from faeces, and tests of individually collected faecal samples from three farms showed greater sensitivity of molecular screening in comparison to coproscopy. The knowledge of species proportion within the sample, together with semi-quantitative evaluation of FEC for each genus/species, adds value to the diagnostic readout and, in our opinion, has the potential to improve current diagnostic approaches. The assays will allow the performance of extensive epidemiological/demographic studies on the major gastrointestinal parasites of sheep and will also add a high degree of precision to anthelmintic efficacy testing protocols.

## Supplementary Information

**Additional file 1: Table S1.** Primers and probes used in two four-plex real-time PCR assays.

**Additional file 2: Table S2.** Evaluation of sensitivity and limit of detection of detection systems in singleplex tested on single template DNA, expressed by Cq values. **Table S3**. Evaluation of sensitivity and limit of detection of multiplexed detection systems tested on single template DNA, expressed by Cq values.

**Additional file 3: Table S4.** Evaluation of infection intensity in 44 faecal samples from three farms (F1, F2, and F3).

## Data Availability

All data generated or analysed during this study are included in this published article. The data sets used and/or analysed during the current study are available from the corresponding author on reasonable request.

## Abbreviations

GIN: Gastrointestinal nematode
FEC: Faecal egg counts
LC: Larval cultures
PGE: Parasitic gastroenteritis
L_3_: Third-stage larvae
*ITS1/2*: Internal transcribed spacers 1, 2
*SSU rRNA*: Small subunit rRNA
*COI*: Cytochrome c oxidase subunit I
gDNA: Genomic DNA
EPG: Eggs per gram of faeces
NIC: Negative isolation control
BHQ: Black hole quencher
M1: Multiplex1
M2: Multiplex2
IAC: Internal amplification control
NTC: No-template control
Cq: Quantification cycle
RFU: Relative fluorescence units
LOD: Limit of detection
SD: Standard deviation
CV: Coefficient of variation
MT-PCR: Multiplexed-tandem PCR
cfDNA: Cell-free DNA
FECRT: Faecal egg count reduction tests
