# Supplementary material for: The identification and semi-quantitative assessment of gastrointestinal nematodes in faecal samples using multiplex real-time PCR assays
Source: Parasit Vectors. 2021 Aug 9;14:391. doi: 10.1186/s13071-021-04882-4 (PMC8351436; doi:10.1186/s13071-021-04882-4)
Supplement: Supplementary file 1 — Additional file 1: Table S1. Primers and probes used in two four-plex real-time PCR assays. [file 13071_2021_4882_MOESM1_ESM.docx]

**Additional file 1: Table S1.** Primers and probes used in two 4-plex real-time PCR assays.

| Target^a^ | Target | Amplicon size [bp] | Forward primer | Reverse primer | Probe |
| --- | --- | --- | --- | --- | --- |
| *Haemonchus* | *ITS2* | 143 | CGTGATGTTATGAAATTGTAAC | CTCAGGTTGCATTATACAAAT | HEX-TGCCACTATTTGAGTGTACTCAGCG-BHQ1 |
| *Teladorsagia* | *ITS2* | 172 | TACTACAGTGTGGCTAACATA | TTCATTGAGTACATTCAAATAGTAG | 6FAM-CCGTCGTAACGTTCCTGAATGATATG-BHQ1 |
| *Trichostrongylus* | *ITS1* | 116 | GCAATAATACCGCCTCATCG | CATAATGGCGTCTAGGCGAG | TxRd-TACGGTACCTGGTTCACAGGAAACC-BHQ2 |
| *Nematodirus* | *SSU rRNA* | 150 | CTCTATGGAAGGTGTCTACC | CTAGTCACCAACGTAAAACAG | HEX-CGGCAGTGAATCGTCGTGCA-BHQ1 |
| *Ashworthius* | *ITS1* | 167 | TCGATAAATGTGACACAAACTTT | GTACGGGATATAATACTTAGTGAAGTA | 6FAM-TGGCGTCATTGAACATGATCATTAAGGT-BHQ1 |
| *Chabertia* | *COI* | 145 | CACAGGTGTTAAGGTTTTTAG | CAACCTAGAATTTGACAATACTAC | TxRd-TTTTTTGTTTACTATTGGTGGTTTAACAGG-BHQ2 |
| IAC | synthetic oligo | 137 | AACCCCTAAACCGGATGATA | GTTTAGAATGTTTTCTCCCGTAC | Cy5-CTCACCTCCCCGCCCAATACTG-BHQ3 |

^a^*Haemonchus* (was designed to cover species *H. contortus* and *H. placei*), *Teladorsagia* (covering *T. circumcincta*, *T. trifurcata*), *Trichostrongylus* (covering *T. colubriformis*, *T. vitrinus* and *T. rugatus*), *Nematodirus* (designed for *N. battus*), *Ashworthius* (designed for *A. sidemi*) and *Chabertia* (designed for *Ch. ovina*).
