# Supplementary material for: The identification and semi-quantitative assessment of gastrointestinal nematodes in faecal samples using multiplex real-time PCR assays
Source: Parasit Vectors. 2021 Aug 9;14:391. doi: 10.1186/s13071-021-04882-4 (PMC8351436; doi:10.1186/s13071-021-04882-4)
Supplement: Supplementary file 3 — Additional file 3: Table S4. Evaluation of infection intensity in 44 faecal samples from three farms (F1, F2, and F3). [file 13071_2021_4882_MOESM3_ESM.docx]

**Additional file 3: Table S4.** Evaluation of infection intensity in 44 faecal samples from three farms (F1, F2 and F3).

| Sample | | Coprological diagnoses | | Molecular diagnoses | | | | | |
| --- | --- | --- | --- | --- | --- | --- | --- | --- | --- |
|  |  | **Strongylide-type eggs** | | **Semi-quantitative estimation of the number of eggs** | | | | | |
| No. | **ID** | **FEC** | **Degree of infection** | **Total EPG** | **Degree of infection** | ***Haemonchus*** | ***Teladorsagia*** | ***Trichostrongylus*** | ***Chabertia*** |
| 1 | F1 | 1720 | high | 5596 | high | 3731 | 1623 | 0 | 242 |
| 2 | F1 | 1220 | high | 1348 | high | 1121 | 189 | 0 | 38 |
| 3 | F1 | 7160 | high | 10477 | high | 8648 | 1751 | 0 | 78 |
| 4 | F1 | 1520 | high | 2425 | high | 1960 | 137 | 0 | 328 |
| 5 | F1 | 260 | medium | 657 | medium | 201 | 93 | 0 | 363 |
| 6 | F1 | 800 | medium | 1125 | high | 958 | 66 | 0 | 101 |
| 7 | F1 | 720 | medium | 4674 | high | 4544 | 23 | 0 | 107 |
| 8 | F1 | 460 (+20, *N. battus*) | medium | 912 | medium | 911 | 1 | 0 | 0 |
| 9 | F1 | 2000 | high | 7187 | high | 6212 | 877 | 0 | 98 |
| 10 | F1 | 700 | medium | 1308 | high | 533 | 451 | 0 | 324 |
| 11 | F1 | 400 | medium | 713 | medium | 551 | 94 | 0 | 68 |
| 12 | F1 | 2220 | high | 6376 | high | 5569 | 717 | 0 | 90 |
| 13 | F1 | 2000 | high | 6903 | high | 5637 | 962 | 0 | 304 |
| 14 | F2 | 240 | medium | 651 | medium | 67 | 543 | 1 | 40 |
| 15 | F2 | 240 | medium | 2735 | high | 2545 | 0 | 138 | 52 |
| 16 | F2 | 240 | medium | 1112 | high | 1036 | 0 | 20 | 56 |
| 17 | F2 | 2640 | high | 11005 | high | 7274 | 2922 | 809 | 0 |
| 18 | F2 | 2020 | high | 902 | medium | 853 | 17 | 32 | 0 |
| 19 | F2 | 140 | medium | 634 | medium | 163 | 425 | 44 | 2 |
| 20 | F2 | 1020 | high | 2062 | high | 1313 | 581 | 24 | 144 |
| 21 | F2 | 0 | - | 0 | - | 0 | 0 | 0 | 0 |
| 22 | F2 | 40 | low | 628 | medium | 231 | 397 | 0 | 0 |
| 23 | F2 | 400 | medium | 2571 | high | 403 | 1765 | 116 | 287 |
| 24 | F2 | 480 | medium | 1224 | high | 359 | 759 | 79 | 27 |
| 25 | F3 | 0 | - | 42 | low | 39 | 0 | 3 | 0 |
| 26 | F3 | 40 | low | 171 | medium | 2 | 168 | 0 | 1 |
| 27 | F3 | 40 | low | 2372 | high | 4 | 2271 | 66 | 31 |
| 28 | F3 | 80 | low | 1079 | high | 134 | 939 | 6 | 0 |
| 29 | F3 | 20 | low | 178 | medium | 160 | 0 | 15 | 3 |
| 30 | F3 | 3020 | high | 8126 | high | 160 | 5776 | 2188 | 2 |
| 31 | F3 | 0 | - | 19676 | high | 14140 | 1005 | 4522 | 9 |
| 32 | F3 | 860 (+80, *Nematodirus* spp.) | medium | 5207 | high | 930 | 1818 | 2410 | 49 |
| 33 | F3 | 20 | low | 41 | low | 0 | 0 | 15 | 26 |
| 34 | F3 | 0 | - | 1001 | high | 71 | 179 | 751 | 0 |
| 35 | F3 | 80 | low | 249 | medium | 245 | 0 | 4 | 0 |
| 36 | F3 | 40 | low | 489 | medium | 0 | 471 | 0 | 18 |
| 37 | F3 | 780 | medium | 622 | medium | 0 | 229 | 387 | 6 |
| 38 | F3 | 100 | medium | 15301 | high | 9564 | 2984 | 2733 | 20 |
| 39 | F3 | 5940 | high | 382 | medium | 69 | 201 | 112 | 0 |
| 40 | F3 | 13640 | high | 15287 | high | 12564 | 190 | 2533 | 0 |
| 41 | F3 | 1200 (+40, *Nematodirus* spp.) | high | 27479 | high | 22256 | 2327 | 2797 | 99 |
| 42 | F3 | 5780 (+120, *Nematodirus* spp.) | high | 3788 | high | 0 | 2806 | 982 | 0 |
| 43 | F3 | 7780 (+120, *Nematodirus* spp.) | high | 109 | medium | 39 | 0 | 6 | 64 |
| 44 | F3 | 12080 (+100, *Nematodirus* spp.) | high | 12646 | high | 5855 | 3355 | 3425 | 11 |

Total strongyle EPG calculated from Cq values resulting from multiplex real-time PCR and the relative proportions of the different nematodes in each sample were compared with the FEC from the faecal samples of the same individuals determined by the Concentration McMaster technique (sensitivity of 20 EPG). For multiplex real-time PCR the amount of 1 EPG corresponding to 234 copies of plasmid standard was considered a minimum limit for positive detection. *Nematodirus* (*N. battus*) and *Ashworthius* (*A. sidemi*) DNA was absent. *Abbreviations:* EPG, eggs per gram of faeces; FEC, faecal egg counts.
